# Prioritization of Zoonotic Diseases in Kenya, 2015

**DOI:** 10.1371/journal.pone.0161576

**Published:** 2016-08-24

**Authors:** Peninah Munyua, Austine Bitek, Eric Osoro, Emily G. Pieracci, Josephat Muema, Athman Mwatondo, Mathew Kungu, Mark Nanyingi, Radhika Gharpure, Kariuki Njenga, Samuel M. Thumbi

**Affiliations:** 1 Global Disease Detection Program, Division of Global Health Protection, United States Centers for Disease Control and Prevention, Nairobi, Kenya; 2 Zoonotic Disease Unit, State Department of Veterinary Services; Ministry of Agriculture Livestock and Fisheries, Nairobi, Kenya; 3 Zoonotic Disease Unit, Department of Preventive and Promotive Heath; Ministry of Health, Nairobi, Kenya; 4 Division of Vector-Borne Diseases, United States Centers for Disease Control and Prevention, Atlanta, Georgia, United States of America; 5 Epidemic Intelligence Service, Center for Surveillance, Epidemiology and Laboratory Services, United States Centers for Disease Control and Prevention, Atlanta, Georgia, United States of America; 6 Field Epidemiology and Laboratory Training program, Ministry of Health, Nairobi, Kenya; 7 Department of Public Health, Pharmacology and Toxicology, University of Nairobi, Kenya; 8 Department of Biomedical Sciences, Colorado State University, Fort Collins, Colorado, United States of America; 9 Center for Global Health Research, Kenya Medical Research Institute, Nairobi, Kenya; 10 Paul G. Allen School for Global Animal Health, Washington State University, Pullman, Washington, United States of America; University of Pretoria, SOUTH AFRICA

## Abstract

**Introduction:**

Zoonotic diseases have varying public health burden and socio-economic impact across time and geographical settings making their prioritization for prevention and control important at the national level. We conducted systematic prioritization of zoonotic diseases and developed a ranked list of these diseases that would guide allocation of resources to enhance their surveillance, prevention, and control.

**Methods:**

A group of 36 medical, veterinary, and wildlife experts in zoonoses from government, research institutions and universities in Kenya prioritized 36 diseases using a semi-quantitative One Health Zoonotic Disease Prioritization tool developed by Centers for Disease Control and Prevention with slight adaptations. The tool comprises five steps: listing of zoonotic diseases to be prioritized, development of ranking criteria, weighting criteria by pairwise comparison through analytical hierarchical process, scoring each zoonotic disease based on the criteria, and aggregation of scores.

**Results:**

In order of importance, the participants identified severity of illness in humans, epidemic/pandemic potential in humans, socio-economic burden, prevalence/incidence and availability of interventions (weighted scores assigned to each criteria were 0.23, 0.22, 0.21, 0.17 and 0.17 respectively), as the criteria to define the relative importance of the diseases. The top five priority diseases in descending order of ranking were anthrax, trypanosomiasis, rabies, brucellosis and Rift Valley fever.

**Conclusion:**

Although less prominently mentioned, neglected zoonotic diseases ranked highly compared to those with epidemic potential suggesting these endemic diseases cause substantial public health burden. The list of priority zoonotic disease is crucial for the targeted allocation of resources and informing disease prevention and control programs for zoonoses in Kenya.

## Introduction

Pathogens of zoonotic origin form two-thirds of all pathogens infectious to man including newly emergent infections [1, 2]. Whereas the public health burden and socio-economic impact of these zoonotic pathogens vary over time and across geographical settings, their impact is often underestimated due to limited surveillance and paucity of disease burden data in most developing countries. In most cases, zoonotic diseases that cause epidemics are better characterized and tend to attract more attention and investments in prevention and control among policy makers nationally and internationally compared to endemic zoonotic diseases that heavily impact rural communities in developing countries [3–5]. For instance, the economic impact of the 2006–2007 outbreak of Rift Valley Fever (RVF) in Kenya was well-characterized and estimated at US$32 million [6], whereas the economic impact of the more commonly occurring endemic zoonotic diseases such as rabies and anthrax remains largely undetermined. For effective management of all zoonotic diseases at the national level in the context of other competing human and animal health threats, prioritization of zoonotic diseases is a key management tool in informing resource allocation.

Several approaches to prioritization of diseases using either qualitative, semi-quantitative or quantitative techniques have been developed [7]. Quantitative methods are best applied where empirical data such as disease burden and socio-economic impact exist, and where there are effective surveillance systems [8–10]. Semi-quantitative and qualitative methods are used where data necessary for prioritization is either insufficient or not available [7, 9]. While selection of the approach to employ differs with the data available, the prioritization process should follow a systematic process that is transparent, replicable and consultative to ensure validity of outcome [7].

The control of zoonotic diseases and events requires close collaboration of human and animal health sectors and their stakeholders in order to effectively and efficiently reduce their emergence and spread [4, 11, 12]. In 2012, Kenya started the implementation of the “One Health” approach through the formation of an inter-ministerial coordination unit referred to as the Zoonotic Disease Unit (ZDU). The mandate of the ZDU is to enhance collaboration between the human, animal, and environmental health sectors for the prevention, control and management of zoonotic diseases [13]. Through the ZDU, Kenya developed a five-year (2012–2017) strategic plan for the implementation of one health in the country and listed zoonotic diseases of importance in Kenya but these were not ranked in order of priority [13]. Here, we conducted a semi-quantitative prioritization of zoonotic diseases ranking them in order of relative importance to guide allocation of resources for development and implementation of prevention and control strategies for these diseases in Kenya.

## Methods

The prioritization of zoonotic diseases was carried out through a facilitated consultative process involving 36 experts in zoonoses from the public health (n = 19), animal health (n = 15) and wildlife health (n = 2), during a three day workshop in September 2015. To select participants, institutions (government, research and academia) and departments that work on zoonoses in areas of surveillance, research and diagnostics on both human and animal health were identified and invitations sent out requesting for experts on zoonoses. Those who were nominated voluntarily agreed to participate. About 20% of the zoonoses experts selected by their institutions had not previously participated in ZDU activities. To minimize systematic bias arising from participants from same institutions ‘thinking the same way’, participants from same institutions and expertise were purposively placed in different groups.

### Selection of prioritization tool

Quantitative data on zoonotic diseases in Kenya is only available for a select few diseases. As a result, a semi-quantitative tool developed by the US Centers for Disease Control and Prevention was used [9]. We made modifications to the One Health Zoonotic Disease Prioritization (OHZDP) tool so that it could be administered to five groups each consisting of 6–7 persons, rather than to a maximum of 12 individuals as previously reported[9]. Each group comprised of two to three public health and animal health professionals working in surveillance, disease control, laboratory, academic and research. The prioritization process consisted of five steps: identification of zoonotic diseases to be prioritized, development of measurable criteria for ranking these diseases, pairwise comparison of the criteria in order to assign weights to each disease, scoring each disease based on the criteria using a decision tree analysis, and aggregation of scores taking into consideration the weighted criteria [9].

### Selection of zoonotic diseases to be prioritized

Starting with a list of 30 zoonotic diseases populated by experts as diseases relevant to Kenya during the formation of the ZDU in 2012 [13], participants reviewed the list to include all zoonotic diseases that are suspected or known to be present in Kenya or the East African region in the last 20 years (1995–2015) since those diseases that may not be present in the country have potential for introduction from the region through travel and trade such as Ebola virus disease. From this process, Q-fever, Middle East Respiratory Syndrome Coronavirus (MERS CoV), chikungunya, taeniasis, sarcopsis, histoplasmosis hantavirus, and Lassa virus were added to the list. In addition, participants included anti-microbial resistance (AMR) pathogens due to the potential transmission of AMR microbes or resistance genes from livestock to humans [11, 14], while tularemia, toxoplasmosis and trichinosis were removed from the list leaving a final list of 36 diseases.

### Selection and weighting of criteria for ranking the diseases

Each group was tasked to come-up with a set of five criteria that were subsequently discussed in a plenary session to produce a combined final list of five criteria that were used to evaluate the diseases, (Table 1). From a list of nine criteria, four criteria were dropped after the plenary discussion to limit the number to the five as suggested by the OHZDP tool [9]. First, ‘possibility of rapid gains following public health intervention’ was deemed to fall in part within the criteria evaluating the potential for effective interventions. Secondly the ‘epidemic potential of zoonotic disease’ was found to be a more robust measure of public health impact than ‘ease of animal to human transmission’ a metric that would assess ease of pathogen transmission from one host to another. Thirdly, for ‘socio-economic, food security and safety’ as a criteria the participants argued that the socio-economic impact would encompass impact of a disease on food security to communities while food safety on the other hand was felt to be of relatively lesser importance. Finally, most of the participants argued that ‘bioterrorism’ is of much lesser relative importance in our setting as opposed to developed countries where there is low burden of zoonotic diseases.

**Table 1:** Ranking criteria, associated weighting for each criteria, and the categorical questions for each criteria and response options used to examine each of the 36 zoonotic diseases selected for prioritization in Kenya.

Criteria (weighted scores)	Question (s)	Responses and categories (score)
Severity of illness in humans **(0.23)**	What is the Case Fatality Rate of the Zoonotic Disease if untreated?	0–5% **(0)**; 6–20% **(1)**; 21–50% **(2)**; >50% **(3)**
	What is the disability weight of the Zoonotic Disease, based on WHO Global Burden of Disease classification?	0.0–0.025 **(0)**; 0.026–0.144 **(1)**; 0.145–0.28 **(2)** >0.283 **(3)**
Epidemic potential of ZD **(0.22)**	Has the Zoonotic Disease caused an outbreak in the last 20 years?	Nationally **(2)**; Regionally* **(1)**; Globally **(0)**
	How many counties are/were affected by the Zoonotic Disease in a year during the last outbreak?	≤5 **(0)**; 6–10 **(1)**; >10 **(2)**
Social Economic Impact **(0.21)**	Does the Zoonotic Disease cause >5% decrease in animal productivity (death, morbidity)?	No **(0)**; Yes **(1)**
	Is the Zoonotic Disease associated with restrictions in trade or free movement of animals or humans?	No **(0)**; Yes **(1)**
Prevalence/Incidence of disease **(0.17)**	What is the Zoonotic Disease prevalence in humans or animals?	<1% **(0)**; 1–5% **(1)**; 5–10% **(2)**; 11–30% **(3)**; >30% **(4)**
	How many counties are affected by the Zoonotic Disease?	≤5 **(0)**; 6–10 **(1)**; >10 **(2)**
Potential for effective intervention **(0.17)**	Are there effective vaccines or treatment measures for the Zoonotic Disease in animals or humans?	Vaccine and treatment **(2)**; Treatment but no vaccine **(1)**; No vaccine and no treatment **(0)**

*Referring to Eastern Africa countries

Using a Microsoft Excel^®^ program from the OHZDP tool by groups, a semi-quantitative analytic hierarchy process was used to assign the most important criteria the highest weight, and the least important criteria the lowest weight [9, 10, 15]. Subsequently, each group ranked the five criteria in order of importance and the group results were combined to produce the overall rank and weight of the criteria. This process assessed the consistency of responses, ensuring adherence to both completeness and transitivity of the group choices for each criteria, which are traditionally used as ‘gold standard’ for rational choice in normative decision theory [7, 9]. A consistency ratio of 0.01 or less was considered satisfactory.

Next, a set of categorical questions against each of the criteria were developed through consensus among the participants. A slight modification from the standard OHZDP tool methods was made in that multiple questions were developed for some criteria. Each of the categorical questions had either binomial (e.g. yes/no) or multinomial answers. The multinomial answers were ordinal in nature with a maximum of 5 categories (e.g. scoring 0–4) for each question used to assess the criterion (Table 1). Where multiple questions existed for one criteria, scores were summed up.

Participants were provided with available epidemiologic data on each of the diseases from both published and unpublished literature including: reported case fatality rate, human morbidity and mortality rates, Disability Adjusted Life Years (DALYs), evidence of sustained transmission in humans, animal disease burden and vector information, availability of effective treatment and vaccines for use in animals and humans and the estimates of the socio-economic impact of the diseases.

To assign each of the diseases a measure of severity of illness in humans, case fatality rate (CFR) in untreated patients and the Disability Adjusted Life Years (DALYs) were used as proxy measurements [16] (Table 1). For diseases that present with multiple syndromes such as RVF, which has mild to hemorrhagic forms, the CFR for the most severe syndrome was considered. The disability weights as presented in the WHO Global Burden of Disease study were used. Diseases with unknown CFRs or DALYs were assigned CFRs or DALYs of diseases with similar syndromes in humans. For example DALYs for the two clinical syndromes of dengue virus infection, dengue fever and dengue hemorrhagic fever were used to assign scores for viral hemorrhagic fevers including RVF, Ebola, Yellow fever and Marburg. DALYs for episodes of malaria was used to assign scores for Q-fever.

Information on zoonotic diseases associated with trade restrictions was derived from a list of transboundary diseases by the World Organization for Animal Health (OIE). This list (list A and B) specifies transmissible diseases with potential for rapid spread across national borders with resultant socio-economic and/or public health consequence and are associated with restrictions to international trade of animals and animal products [17]. Diseases on this list such as RVF, anthrax and Q-fever were scored ‘yes’ while those not on the list such as Ebola, WNV and CCHF were scored a ‘no’ on the question on restrictions in trade or free movement of animals or humans.

### Ranking and aggregation of scores for the zoonotic diseases

Using a decision tree approach described in the OHZDP, each group then proceeded to score each zoonotic disease independently based on the answers to the categorical questions for each weighted criterion [9]. Where country level data were not available, data from the East Africa region was used. In the absence of any published data from Kenya and the region, expert opinion was used to assign a disease to a level. This process was repeated for all 36 diseases on the list.

For each disease, the weighted scores for each criteria were summed to obtain a total weighted score by group. Subsequently an average weighted score (from all five groups) was obtained and normalized in relation to the maximum score, yielding a normalized final score within a range of 1 to 0 that was used to rank the diseases. This was a modification from the standard OHZDP tool method where facilitators assign scores for each disease in one spreadsheet and the individual weighted scores are aggregated and normalized [9] Thereafter, the ranked disease list was reviewed during a plenary session.

### Sensitivity analysis

To assess the robustness of the prioritization outcome, variability in weighting of the criteria, consensus building in groups and expert opinion scoring of disease data were evaluated. First, the five criteria were given equal weights of 1 to obtain normalized scores for each disease as described [9, 18]. Next, each of the five criteria was systematically removed from the process to obtain normalized scores for each disease. Finally, each of the five groups was removed to assess the impact each group had on the final normalized scores. Pearson’s product-moment correlation was used to assess the relationship between normalized scores obtained using the OHZDP tool that produced the ranked priority disease list reported here and the adjusted scores, assessing impact of criteria weight and contribution of each criteria and group respectively. Pearson’s correlation coefficient was considered significant at p-value <0.05.

Ethical approval was not sought since the activity was not human subject’s research, the primary intent was public health practice using data that is publicly available. Informed consent was not sought from the participants and all data was analyzed anonymously.

## Results

The final list of diseases to be prioritized included 36 diseases/pathogens. The distribution of calculated weight and rank for each of the five criteria from each of the five groups is given in Table 2. The criterion evaluating illness in humans was ranked highest in three of the five groups and second in the remaining groups and ranked highest overall.

**Table 2:** Ranking of criteria using analytical hierarchical process: criteria weight and rank for each of the groups.

Group	Group 1	Group 2	Group 3	Group 4	Group 5	Overall ranking
Severity of illness	0.43–1	0.24–2	0.49–1	0.35–1	0.28–2	1
Epidemic potential	0.28–2	0.44–1	0.19–3	0.25–2	0.14–3	2
Socio-economic impact	0.17–3	0.10–4	0.07–4	0.21–3	0.48–1	3
Prevalence of disease in humans or animals	0.07–4	0.04–5	0.21–2	0.03–5	0.06–4	4
Interventions	0.05–5	0.18–3	0.04–5	0.16–4	0.05–5	4
Consistency ratio*	0.06	0.1	0.07	0.05	0.07	-

*A consistency ratio < 0.1 is acceptable

Overall, the top five priority diseases in descending order were anthrax, trypanosomiasis, rabies, brucellosis and RVF (Table 3). Viral and bacterial zoonoses made up 60% of zoonotic pathogens at 36.1% and 25% respectively; zoonoses caused by helminths were 13.9%, protozoan and fungi 8.3% each, ecto-parasites 5.5% and others 2.8%. Overall, zoonotic diseases with limited data including West Nile virus fever, Lassa fever, diphyllobothriasis or no local or regional data including, hantavirus fever, and histoplasmosis generally ranked lowest since two of the criteria, epidemic potential and prevalence of disease relied on presence of local data.

**Table 3:** Ranked Priority disease list for Kenya with ranking by criteria and normalized final scores, 2015.

Disease	Overall ranking by criteria					Normalized Final scores
	Severity of illness	Epidemic potential	Socio-economic impact	Prevalence of disease in humans or animals	Available Intervention	
Anthrax	5	2	1	4	1	1
Trypanosomiasis	1	4	1	9	3	0.94
Rabies	4	5	3	5	1	0.93
Brucellosis	9	4	1	1	2	0.89
Rift Valley fever	9	1	1	2	5	0.87
Echinococcosis (Hydatidosis)	7	7	6	3	3	0.73
Non Typhi Salmonellosis	12	2	6	7	1	0.7
Q fever*	11	6	5	1	4	0.69
Mycobacterium spps	7	6	2	8	7	0.67
Influenza and pandemics	8	8	2	9	6	0.64
Cysticercosis	12	8	4	5	3	0.62
Dengue	7	2	7	3	10	0.6
Leptospirosis	7	8	4	5	3	0.6
Schistosomiasis	11	8	9	3	3	0.58
Yellow fever	6	10	5	11	5	0.54
Rickettsiosis	10	8	10	6	3	0.52
Taeniosis*	14	9	7	5	3	0.51
Sarcopsis*	14	11	5	7	3	0.5
Cryptosporidiosis	13	9	8	4	4	0.49
Leishmaniasis	7	8	9	10	6	0.49
Ebola	2	14	4	13	9	0.48
Marburg	3	14	5	13	10	0.42
Crimean-Congo hemorrhagic fever	5	11	6	9	10	0.42
Antimicrobial resistance*	14	3	7	9	8	0.42
Dermatophylosis	14	12	9	9	3	0.36
Cryptococcosis	12	12	10	9	4	0.36
Listeriosis	12	14	5	12	5	0.35
Aspergillosis	12	13	9	11	5	0.34
MERS-CoV*	14	16	6	8	10	0.34
Plague	14	15	9	13	6	0.32
Chikungunya*	10	9	9	8	10	0.31
West Nile Virus	10	12	9	10	10	0.24
Histoplasmosis*	13	15	10	13	5	0.22
Diphyllobothriosis	14	17	9	13	4	0.19
Hanta virus fever*	8	17	8	13	10	0.17
Lassa fever*	9	17	9	13	10	0.13

* Zoonotic diseases that were newly added to the list in 2015

For severity of illness, diseases with a CFR of >50% such as Ebola, Marburg, human African trypanosomiasis (HAT) and rabies were scored highly and diseases/events with a CFR ≤ 5% including RVF, brucellosis, salmonellosis and AMR scored lower. While pathogens classified as viral hemorrhagic fevers were generally scored higher on adjusted disability weights, HAT, Ebola, rabies and yellow fever were assigned the highest score by three or more groups.

In the assessment of epidemic potential, endemic zoonotic diseases that are reported in humans and animals annually in multiple counties in Kenya through the Ministry of Health’s Integrated Disease Surveillance and Response (IDSR) System and the Directorate of Veterinary Services surveillance system were assigned high scores. This includes diseases such as anthrax, RVF, rabies, Q-fever, dengue fever and brucellosis.

High scores were assigned to anthrax, brucellosis, RVF and *Mycobacterium* species, diseases for which outbreaks in livestock are associated with high direct and indirect losses in productivity and market losses associated with quarantines and trade bans. Diseases that had reported high prevalence and in multiple counties including brucellosis, schistosomiasis and RVF were assigned high scores while diseases that had not been reported in Kenya or reported less than 1% prevalence in less than two counties including a number of viral hemorrhagic fevers (Ebola and Marburg), plague, Lassa fever, hantavirus and fungal diseases including histoplasmosis and cryptococcosis were assigned a score of 0.

Anthrax, brucellosis and most of the bacterial infections were scored highly for potential for intervention since vaccines and treatments are available for humans or animals. However, most of the viral diseases with the exception of RVF, yellow fever and influenza scored low in this category due to the unavailability of vaccines and drugs.

### Sensitivity analysis

**Fig 1:**
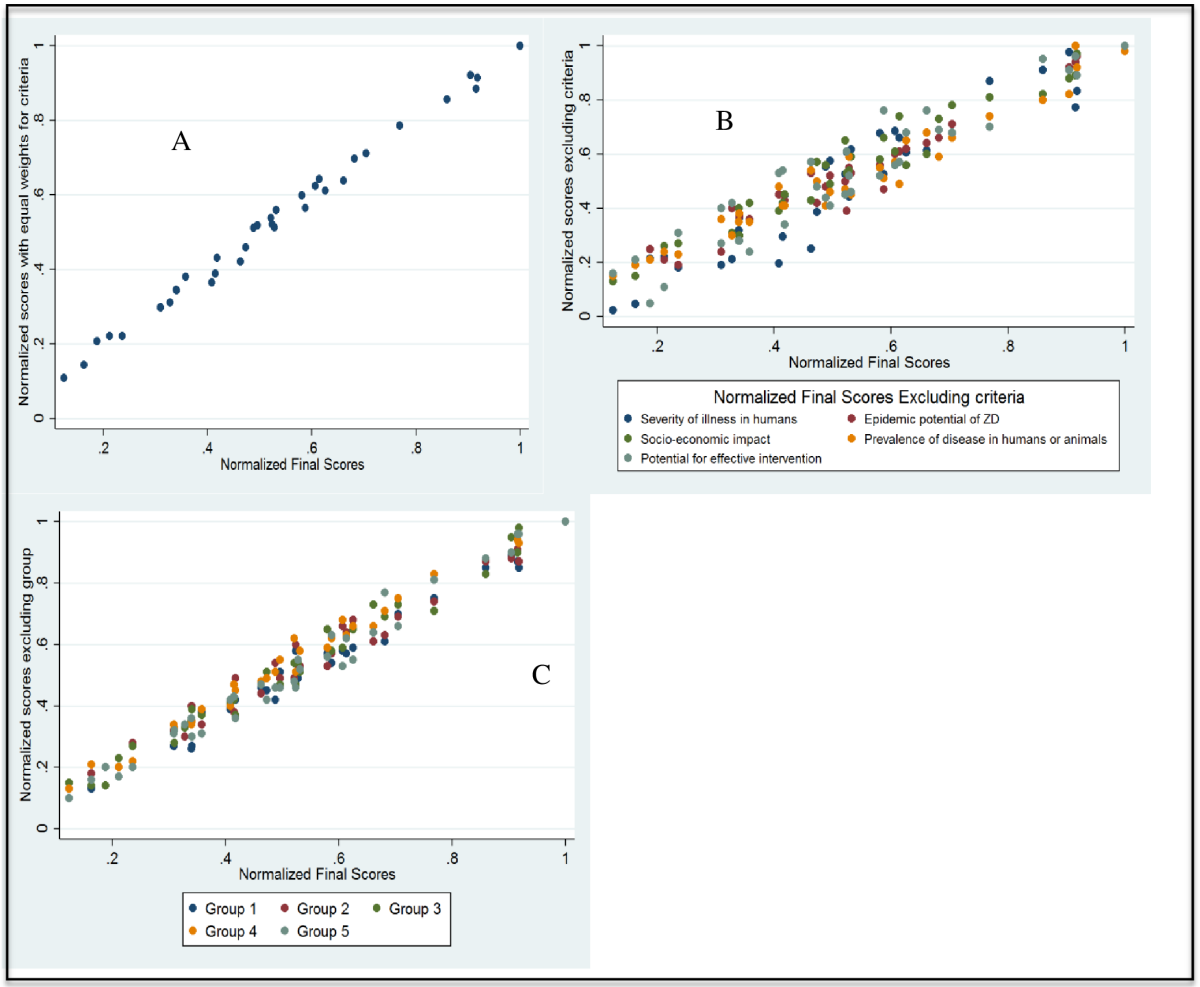
Comparison of normalized scores obtained from the weighted criteria and (a) equal weights; (b) excluding each of the five criteria and (c) excluding each of the five groups.

There was a strong positive correlation between normalized scores and adjusted scores, produced by the OHZDP tool, seen when comparing weighted and unweighted criteria [r (34) = 0.99, p < 0.05], when excluding each criteria from the model [r (34) (0.94–0.98), p < 0.05], and when excluding each groups from the model [r(34) (0.94–0.98) p < 0.05], Fig 1.

Specifically, there were minimal changes in disease ranking for the first five diseases on the final ranked disease list except when severity of illness in humans was excluded, where trypanosomiasis dropped three levels (ranked 6^th^) and non-typhi salmonellosis moved up two levels (ranked 4^th^). Anthrax was ranked first regardless of weight of criteria and group contribution and second when prevalence of disease in humans and animals was excluded.

## Discussion

The top five diseases identified as priority zoonotic diseases in Kenya were anthrax, trypanosomiasis/HAT, rabies, brucellosis and RVF in descending order. Overall, neglected zoonotic diseases ranked highly, highlighting the high burden and importance of non-epidemic diseases in the local context. Among the top ten priority diseases in Kenya, five diseases (anthrax, trypanosomiasis, rabies, brucellosis, and hydatidosis) have been listed as neglected zoonotic diseases by WHO [5]. This highlights the importance of prioritizing diseases at country level as it presents the opportunity to focus on diseases that have the greatest public health burden and not just diseases that have greater global attention as the epidemic prone diseases.

It has been suggested that neglected zoonotic diseases, which primarily impact poor rural communities, are in part neglected due to underreporting and underestimation of disease burden resulting from use of unreliable disease metrics extrapolated from scanty data [4, 19]. This results in the systematic underweighting of these diseases and lower investments in prevention and control programs by health authorities compared to emerging diseases with epidemic potential that attract the attention of policy and political decision makers globally and in the developed countries [4, 12, 19]. For example, in North America and Japan, viral hemorrhagic fevers (Ebola and Marburg virus hemorrhagic fever and Lassa fever), epidemic diseases (Influenza (H1N1) and severe acute respiratory syndrome), rabies, Nipah virus encephalitis and prion diseases (variant Creutzfeldt-Jakob disease and Bovine spongiform encephalopathy) were among the top five priority diseases, reflecting the focus on risk-based priority setting to zoonotic diseases with high impact on public health and trade [9, 19]. Many of the endemic diseases in Kenya are absent or low in prevalence in many of the developed countries further reducing attention given to them. However, similar to Kenya the disease priority list in the Netherlands was mostly endemic diseases including *Toxoplasma gondii*, campylobacter species and *Coxiella burnetti* [18]

The prioritization work provided an opportunity to update the zoonotic disease list with inclusion of newly reported zoonotic diseases/events in Kenya such as MERS CoV and antimicrobial resistance, an emerging global concern that is driven in part by transmission of drug-resistant pathogens from livestock to humans through use of antimicrobials to maintain animal health and increase livestock productivity [10]. However, this list should not be static and should be reviewed regularly to take account of new data arising including of emerging infections such as ZIKA virus currently receiving attention across countries and diseases that are could have been overlooked such as toxoplasmosis. In addition, in line with the goal of this exercise and to identify and prioritize endemic zoonotic diseases with higher health burden locally, higher weights were assigned to diseases with larger geographical occurrence nationally as opposed to diseases that have occurred regionally or globally. Majority of emerging diseases that have occurred globally or regionally such as Ebola hemorrhagic fever, highly pathogenic avian influenza and Zika often receive funding from international agencies for preparedness and response based on risk of occurrence and at times irrespective of local occurrence. So it very likely in another setting depending on the goal of the prioritization exercise for these major zoonotic diseases that have occurred globally to be scored higher. The prioritized disease list provides a basis for the design of prevention and control programs for zoonoses, and allocation of resources to enhance zoonotic disease management in Kenya.

The OHZDP tool and similar approaches have been used previously using varied criteria and different stakeholders [8–10, 18, 20, 21]. Use of this tool allowed for multi-stakeholder groups to provide input from a broad base of experience for ranking of criteria and the consultative nature would enhance buy-in of the final list of prioritized diseases by different sectors in the country for future allocation of resources in zoonotic disease management programs. The sensitivity analysis demonstrated that the weight of the criteria, the criteria used, and individual groups did not have significant bias in our final ranking of the prioritized disease list. This could likely be due to the systematic use of data provided for the groups, similar nature of the criteria and questions developed by the groups, or an existing collaborative or shared vision of the participants that made this slightly modified version of the OHZDP tool appropriate for zoonotic disease prioritization in Kenya.

There were however, several limitations to this work. First, the decision tree analysis requires metric measurements of disease occurrence, which were lacking for a number of diseases or that were only available from limited studies that may not be representative of the entire country. In addition, some of the disease metrics such as prevalence, OIE classification of diseases and case fatality rates could not adequately evaluate all the diseases being prioritized such as AMR. In cases where disease data were unavailable, experts provided estimates based on data from the region or from diseases closest in epidemiology to those being examined hence introducing bias. Use of multiple groups in assigning scores from expert opinion partly mitigated subjective bias that is inherent in semi-quantitative scoring. Secondly, it has been recognized that disease metrics such as DALYS that have been generated with data largely from the developed world could underestimate the public health burden of neglected endemic zoonotic diseases. Thirdly, we used a slightly modified version of the OHZDP tool, hence our output may not be comparable to outputs from other countries that used the standard version; additionally this modified approach may not be appropriate in all settings, especially when individual over group opinion is needed for buy-in and consensus building. Finally, the epidemiology of certain diseases in Kenya could have resulted in overestimation of true burden in the country resulting in high ranking of these diseases. For example, HAT is localized in western parts of Kenya while trypanosomiasis in livestock caused by *Trypanosome* species that are largely non-zoonotic is associated with high economic impact that resulted in high ranking of this disease despite the fact no HAT case had been reported in the last decade. Similarly, outbreaks of anthrax are associated with dramatic clinical disease events that render them easier to report and document.

Application of this modified version of the OHZDP tool allowed us to use qualitative and quantitative data to generate metrics for ranking diseases in a larger group setting. It is important to point out that while this disease list could be used to guide the allocation of limited resources to control diseases that ranked highly, areas of collaboration in surveillance and research should be explored for all diseases since it’s likely that the lack of data particularly for diseases such as Hanta virus fever and histoplasmosis, that no surveillance programs are in place could have influenced the final outcome. Investments in innovative collaborative multi-pathogen surveillance and research programs to generate disease prevalence and burden data, and development of effective disease prevention and control strategies in human and animal populations are some key recommendations to enhance future prioritization exercises. The methods used here could potentially be applied across different health sectors to rank public health needs.
